# Use of Nanoparticles for Enhancing the Interlaminar Properties of Fiber-Reinforced Composites and Adhesively Bonded Joints—A Review

**DOI:** 10.3390/nano7110360

**Published:** 2017-11-01

**Authors:** Davide De Cicco, Zohreh Asaee, Farid Taheri

**Affiliations:** Advanced Composite and Mechanics Laboratory, Department of Mechanical Engineering, Dalhousie University, 1360 Barrington Street, P.O. Box 15 000, Halifax, NS B3H 4R2, Canada; davide.decicco@dal.ca (D.D.C.); zohreh.asaee@dal.ca (Z.A.)

**Keywords:** delamination enhancement, nanoparticles, composite materials, adhesively bonded joints, interlaminar strength

## Abstract

This review paper aims at reporting some of the notable works carried out concerning the use of nanoparticles (NPs) as a means of improving the resistance of fiber-reinforced polymer composite materials (FRPs) and adhesively bonded joints (ABJs) to delamination initiation and propagation. Applications of various nanoparticles, such as carbon-based, ceramic-based and mineral-based are discussed. The main properties that have been considered for improving the delamination and fatigue resistance of FRPs are the interlaminar shear strength, fracture toughness, and fracture energy. On the other hand, cohesive and interfacial strengths have been the focused parameters in the works that considered enhancement of ABJs. The reported results indicate that inclusion of NPs in polymeric matrices leads to improvement of various material properties, even though some discrepancies in the results have been noted. Notwithstanding, additional research is required to address some of the issues that have not yet been tackled, some of which will be identified throughout this review article.

## 1. Introduction

The excellent mechanical properties, tailorability, and remarkable resistance to corrosion and fatigue of fiber-reinforced polymer composite materials (FRPs) have attracted growing interests from automobile, aeronautical, marine, and construction industries. However, despite their great attributes, the relatively inferior interlaminar properties of FRPs have prevented the growth of their applications in the way they deserve. The interlaminar inferiority is the Achilles-heal caused by the relatively low strength and brittle nature of the resins that are commonly used to form a great majority of structural FRPs. Not only the weakness impacts the satisfactory performance of FRPs in some applications, but it also significantly affects the performance of adhesively bonded joints (ABJs).

**Figure 1:**
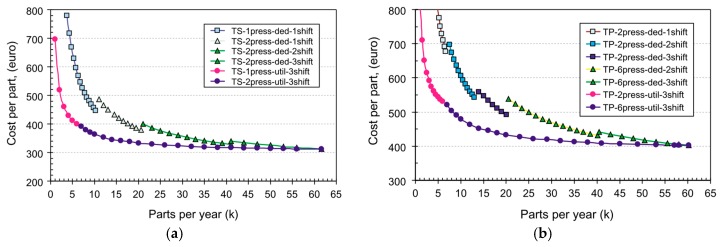
Comparison of production costs for manufacturing a floor pan made of (**a**) thermoset resin and (**b**) thermoplastic resin [6] (Reprinted from Compos. Part A Appl. Sci. Manuf., 37, Verrey, J.; Wakeman, M.D.; Michaud, V.; Månson, J.A.E., Manufacturing cost comparison of thermoplastic and thermoset RTM for an automotive floor pan, 9–22, (2005), with permission from Elsevier).

The most widely used class of polymers for producing fiber-reinforced structural load bearing components is thermoset resins. Compared to their thermoplastic counterparts, they are generally stiffer and stronger and more cost-effective. In addition, the overall production cost of thermoplastic composite parts is higher than that of thermosets, as can be seen from the data illustrated in Figure 1. Comparatively, the raw material and tooling costs are also higher for thermoplastic resins, and their prepregs [1]. However, thermoset resins are comparatively more brittle, as can be seen from the data reported in Table 1 (see for example, properties of epoxy and polyether-ether-ketone). Due to this brittle nature, fiber-reinforced thermoset composites tend to be more prone to interlaminar damage and delamination [2]. This mechanism also causes issues in fiber metal laminates (FMLs), causing delamination of their metallic sheets from their neighboring fiber-reinforced laminates. In general, delaminations originate due to interlaminar stress concentrations in the vicinity of cut-outs and sudden ply drop-offs, or due to impacts (could be caused by a meager impact caused by a tool drop), and due to inadvertent issues occurring during their fabrication (e.g., oily residue left on fibers/fabrics prior to impregnating them with resins). In addition, large loading magnitudes applied on laminates that have a large mismatch in their plies Poisson’s ratio, and repeated cyclic loads can also cause delaminations. In contrast, metals do not exhibit delamination and are, therefore, still the material of choice for certain specific applications. Therefore, inhibiting the onset of delamination, and/or decelerating the propagation rates of delaminations are paramount in extending the service lives of FRPs, FMLs, and ABJs, thereby promoting their wider applications.

Interlaminar shear stresses are the major cause of delamination in FRPs and ABJs [3]. Therefore, improvements of the interlaminar shear strength (ILSS), fracture toughness (ILFT) and fracture strength (ILFS) would all help to mitigate the onset and propagation of delaminations [4]. One of the most effective means to enhance the interlaminar properties of polymer resins can be attained by inclusion of nanoparticles in such resins. The present review paper, therefore, aims at providing the reader with an overview of the works that have focused on enhancing the properties of thermoset resins that would in turn improve performance of FRPs, FMLs, and ABJs under various loading conditions.

It should be noted that improvement of performance of ABJs by such a means is an important aspect of this review article, since ABJs are being widely used to either assemble structural components, or to facilitate speedy and cost-effective repair and rehabilitation of various structures. Compared to mechanical fastened joints, ABJs are relatively lighter, and when designed optimally, they result in joints with comparatively lesser stress concentrations and much-improved fatigue lives. While both thermoset and thermoplastic resins are being used to form ABJs, both resins have their own issues. The main issue of thermoset resins/adhesives (i.e., their brittle nature) was briefly discussed earlier. In comparison, thermoplastics have a more ductile behavior and higher fracture toughness, but they have lower stiffness, require higher curing temperatures, and are generally more expensive. Due to environmental concerns, the recyclability of thermoplastics has made their use more popular in some applications. Nanoparticles, being relatively extremely stiffer and stronger than both resin types, could provide effective remedies for enhancing the low stiffness and fracture toughness of thermoplastics, as well as improving their bonding strength to substrates, in turn enhancing the reliability of ABJs formed by the resins. This article presents first a brief introduction to the methods that have been developed for improving the interlaminar properties of composites and ABJs. Then, the most commonly used nanoparticles are briefly introduced. This is followed by a detailed description of the effects of the nanoparticles on the interlaminar response of FRPs and ABJs. Finally, the numerical techniques used for modeling the effect of nanoparticles in improving the properties are reviewed. The use of the techniques in various modeling scales will be highlighted. It will be shown how these techniques and their combination have facilitated a thorough understanding of the various mechanisms that have led to improvement in properties of polymeric resins and their composites.

### 1.1. Interlaminar Properties Improvement Techniques Not Using Nanoparticles

Extensive research has been carried out by several researchers with the aim of improving the out-of-plane mechanical properties of laminated composites and adhesively bonded joints, with particular attention given to the properties that mitigate delamination.

Examination of the literature has revealed that mechanical techniques have mainly been used to improve the through-thickness strength of FRPs. For instance, sewing-like techniques, such as 3D weaving, stitching, braiding, and embroidery [7,8,9,10,11,12], have been extensively employed to achieve significant improvement in the interlaminar properties. Improvements in interlaminar shear strength and toughness, delamination resistance, and notch sensitivity have also been reported. However, even though the out-of-plane properties have been shown to have improved, a reduction in the in-plane mechanical properties resulting from the use of such techniques has also been reported by some investigators [11,13]. The resulting issue has been primarily attributed, in part, to fiber misalignment caused by the stitching and weaving processes. Accumulation of resin pockets in the stitching zones has also been considered as a cause of the degradation, because these zones become more prone to generation of microcracks, especially during the curing process. Also, undulation of longitudinal fibers due to placement of transverse fibers has been hypothesized to cause degradation of the tensile and compressive stiffness and strength. Specifically, under a compressive loading, the undulated fibers would have a higher tendency to buckle. The manufacturing cost could also become of concern when considering some of these techniques. Very similar to the noted techniques is the use of z-pins, by which short and thin metallic or composite pins are anchored in a rivet-like fashion, aiming at keeping the different plies together under loading [14,15]. Several techniques for improving the out-of-plane properties of resins (primarily for improving mode I toughness), have also been reported. However, the loss of in-plane properties, similar to that discussed previously, was reported as a result of incorporation of the latter techniques [13,16,17]. The damaging mechanisms resulted in these techniques have been attributed to deviation of fibers, generation of resin-rich pockets, and fiber crunching.

Regarding FMLs, various methods, some of which are reported in Table 2, have been investigated in order to improve the interface strength of their metallic layers. The simplest approach, which is still widely used, is using an optimal surface preparation procedure, mainly done by mechanically abrading the adhering surfaces, using either sandpaper or grit blasting [18,19,20,21]. This method relies on the creation of uniform surface micro-irregularities that would enhance the mechanical grip of adhesives. The creation of micro-pores on the mating surfaces via chemical reaction has been shown to be another viable technique. For instance, Zhao et al. [22] created a porous thin alumina layer on aluminum sheets that would allow polymer molecules to have more surface area to interact with, and therefore, adhere more strongly to the aluminum substrate [23].

Other tried methods involve modifying the chemistry of the surface, in order to facilitate enhanced chemical bonds. The most common ones are plasma activation [24,25,26,27,28,29,30,31,32,33,34,35] and chemical etching [36,37,38,39]. Plasma activation entails bombarding substrates’ surfaces with high-energy plasma, thereby removing impurities and chemically changing the molecular construction of the surface. Chemical etching is another process by which a very thin layer of material is removed from a surface to obtain a non-contaminated area to host the adhesive. The chemical can be tailored such that, after the treatment, the surface remains covered in molecules that are able to create strong bonds with both the adhesive and the metallic substrate.

Despite the availability of the above effective techniques, the issue of relatively weaker interlaminar strength of laminated composites remains as a challenge, as techniques such as stitching cannot be applied to most FRPs in practical cases.

Other methods used for improving the interlaminar properties of FRPs and ABJs are (i) the interleaving method [40,41,42,43,44,45,46,47], by which the addition of micro-size particles are added to resins as reinforcement [48,49] and (ii) modification of the chemistry of resins/adhesives (epoxy, methyl methacrylate, acrylic, etc.) to promote better fiber/matrix interaction [50]. For instance, Lu et al. [47] inserted a thin polyethylene layer in between the plies of a carbon-fiber/epoxy (CF/EP) laminate and observed a reduction in the extent of delamination during an impact event. Taheri [48] showed that inclusion of 1 wt % of silicon carbide whiskers could significantly improve the shear strength and energy absorption capacity of ABJs, especially at sub-freezing temperatures. However, the author reported that cracking of the whiskers led to brittle fracture, thus limiting the effectiveness of the particles.

It is worth mentioning that the interlaminar properties of laminated composites can be optimized, up to a certain degree, with appropriate ply sequencing [51]. Effective ply sequencing can minimize the mismatch of Poisson’s ratio, the coefficient of mutual influence and thermal residual stresses, thereby reducing the magnitude of interlaminar stresses [52]. It should, however, be noted that such theoretically obtained enhancements can at times be easily overbalanced by the technical challenges encountered when fabricating the resulting optimized FRPs; that is because such an optimized composite would often have unconventional ply fiber-orientations.

More recently, a new approach has been tried by several investigators, entailing incorporation of nanoparticles in resins/adhesives used to produce laminated composites and ABJs [53,54,55,56,57,58]. Gains in the interlaminar properties and bonding strength, without compromising the other primordial mechanical properties, have been reported. Details of such optimization are provided in a subsequent section.

### 1.2. Brief Introduction to Nanoparticles

Nanoparticles are microscopic particles with at least one of their dimensions being less than 100 nm in size. NPs come in different compositions, shapes, sizes, and aspect ratio. Their primary role is to act as a bridge within atomic or molecular structures of their host bulk materials. In the case of bulk materials, their physical properties are considered constant, independent of their dimensions. However, nanoscale particles have larger specific surface area, higher surface energy, and a reduced number of structural imperfections, enabling them to exhibit improved properties compared to their properties in their bulk state. Moreover, besides the size effect, the properties are highly dependent on the atomistic configuration, especially the interaction with the surrounding material. This complexity in behavior should be taken into account when considering nanoparticles [60].

Due to the greater mechanical properties of materials exhibited in their nanoscale forms in comparison to their macroscale forms, various types of NPs generated from diverse materials have been introduced. One of the most common types is carbon-based NPs. Based on their shapes, carbon nanofillers are categorized within three different groups; they are: (i) zero dimensional or spherical fillers; (ii) one-dimensional or cylindrical particles; and (iii) two-dimensional particles. The examples for each of these three groups are, respectively: (i) nanodiamonds (NDs); (ii) carbon nanotubes (CNTs) and carbon nanofibers (CNFs); and (iii) graphene nanoplatelets (GNPs). Earlier studies revealed that inclusion of carbon-based NPs (CNTs, CNFs, and GNPs) in polymers improved various properties (e.g., mechanical, electrical, and thermal) of their host polymers [61,62,63,64,65,66,67,68].

Various metals have also been used as nanoparticles; examples are nano-gold, nano-silver, and iron oxide. The effects of inclusion of this group of nanoparticles into a polymer matrix have been shown to increase the host materials’ electrical, optical, magnetic, and mechanical properties, with their applications being mainly in medical and biomedical sciences [69,70,71].

Nanoclays and nanosilicas are mineral type NPs, which are relatively very inexpensive. Nanoclays have been shown to improve the mechanical properties, fire retardancy, and liquid infusion of their host polymers [72,73,74], while addition of nanosilicas to polymers has been shown to enhance the strength, flexibility, and durability of polymers [75,76,77]. Furthermore, polymers themselves can be used in a nano-fiber form, as reported in [78,79]. Note that all the previously cited nanoparticles can also be functionalized; that is, the atomic configuration of their surfaces could be modified, so that stronger links could be created with the surrounding environment, thereby elevating the degree of enhancements.

### 1.3. Mode I and II Fracture Testing Methods

**Figure 2:**
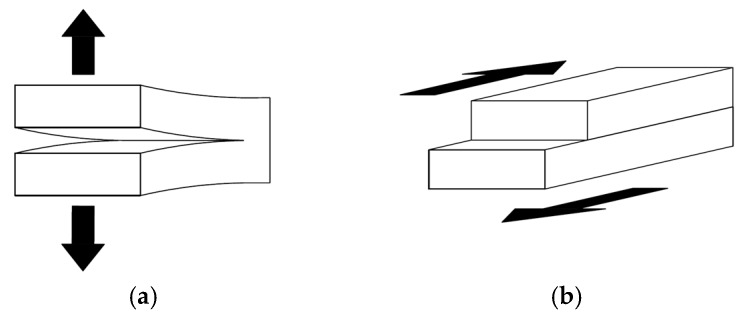
Schematic representation of (**a**) mode I and (**b**) mode II fractures.

**Figure 3:**
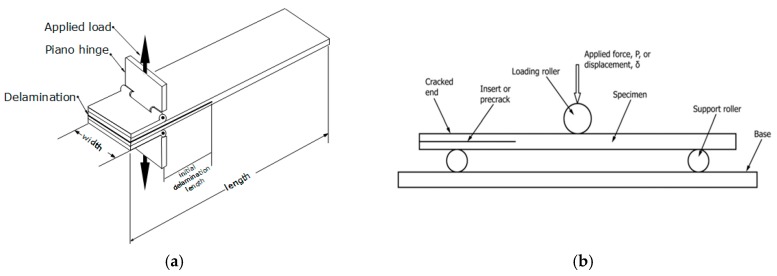
Specimens and loading configurations for (**a**) mode I [80] and (**b**) mode II [81] fracture tests (Reproduced, with permission from ASTM D5528-13 Standard Test Method for Mode I Interlaminar Fracture Toughness of Unidirectional Fiber-Reinforced Polymer Matrix Composites and ASTMD7905-14 Standard Test Method for Determination of the Mode II Interlaminar Fracture Toughness of Unidirectional Fiber-Reinforced Polymer Matrix Composites, copyright ASTM International, 100 Barr Harbor Drive, West Conshohocken, PA 19428).

Tensile and shear mode interlaminar fractures, also referred to as mode I and mode II fractures, respectively, are schematically illustrated in Figure 2. A common testing procedure for characterizing mode I ILFT of fiber-reinforced composites is presented in ASTM D5528 standard [80]. The test specimen (see Figure 3a) consists of a double cantilever beam made of unidirectional plies bonded together, with an intentional delamination created in the mid-plane section, at one end of the specimen. Two piano hinges, or loading blocks, are mounted on the external surfaces on the end portion of the specimen (i.e., at the delaminated region), to facilitate tensile loading of the specimen, by which the delaminate region is pulled apart. The interlaminar fracture toughness value can then be calculated from the force-displacement curve and crack advancement history data traced during loading of the specimen.

Considering mode II (shear) characterization, a common test is ASTM D7905/D7905M [81], which makes use of an end-notched flexure specimen. The schematics of the specimen and loading configurations are illustrated in Figure 3b. Similar to the mode I specimen, two rectangular beams are bonded together, with an intentional delamination created over a defined portion at mid-plane of the specimen. Three-point bending is used to generate sliding (shear) forces at the interface zone, thereby propagating the delamination. The force and delamination extension length are traced and used to calculate the value of the mode II fracture toughness.

## 2. Effect of Nanoparticles on Delamination-Related Properties and ABJs

This section is intended to provide an overview of some of the works that have investigated the effect of inclusion of nanoparticle in FRPs and ABJs with the aim of strengthening their interlaminar properties. The properties include the interlaminar shear strength (ILSS), modes I and II fracture toughnesses and corresponding strengths (ILFT, ILFS), as well as crack propagations under static, dynamic and fatigue loadings. All these parameters have been shown to influence the delamination response of FRPs, and performance of ABJs. It should be emphasized that to take advantage of the enhancement that could be attained in strengthening and toughening resins and adhesive with the use of NPs, it must be ensured that appropriate provisions have been taken to ensure that the interface failure would occur in a cohesive mode. It is only under that condition that the full potential of these NPs could actually be harnessed. This proviso highlights the critical importance of surface morphology and preparation processes.

### 2.1. Enhancement of Interlaminar Shear Strength

The interlaminar shear strength (ILSS) is one of the most influential parameters that governs the delamination resistance and bond-strength. The effects of nanoparticles on ILSS have been studied extensively, as evident by the available relevant literature. The NPs that have been investigated include CNTs, GNPs, multi-walled CNTs (MWCNTs), CNFs, silica and alumina, as well as a few other types of NPs. All the reviewed articles have reported an increase of ILSS with no degradation of the other mechanical properties.

Chandrasekaran et al. [53] carried out an investigation into the effect of the addition of 0.5 wt % of MWCNTs on the ILSS of CF/EP composites, using the compression shear test method. A 41% increase in the ILSS was observed for the non-functionalized particles, while a 61% improvement was obtained with NPs that were functionalized. By comparing the effect of the nanoparticle on the fracture toughness of the matrix and the overall performances of their FRP, the authors concluded that the improvements were due to the chemical interlocking between the fibers and the NPs (with a stronger interlocking in the case of the functionalized NPs), rather than by improving the properties of the matrix itself. Liu et al. [82] observed different outcomes when used both MWCNTs and a reactive aliphatic diluent named n-butyl glycidyl ether (BGE), to enhance the ILSS properties of GF/EP laminates. The MWCNTs proved to enhance the properties of the matrix via crack bridging phenomenon, while the BGE enhanced the matrix/glass-fibers interface adhesion. The combined mechanisms produced a 25% improvement in the ILSS of their FRP. Other methods have also been developed and used for the improvement of ILSS. For instance, Fan et al. [83] managed to disperse MWCNT particles in a preferential direction. The authors developed a technique facilitating the particles’ alignment in the transverse direction with respect to the shear load, while contemporarily reducing the effects of compaction. A 33% improvement in the ILSS was observed with 2 wt % of NP content (with respect to the resin’s weight). Nanoparticle contents higher than 2 wt % led to a mix too viscous to properly wet the fabric. Similarly, Wichmann et al. [84] used the electrical properties of the MWCNT to orient them transverse to the shear direction. Another method for a preferential disposition of CNT was proposed by Abot et al. [4]; the authors grew MWCNT NPs vertically on the surface of a thin fabric, then placed them into a layer of fresh resin, and subsequently deposited the combination in between the plies of CF/EP laminates. While significant improvement in delamination mitigation was expected by the authors, only a slight increase in ILSS was observed.

Graphene nanoparticles have also been shown to be very effective in improving the interlaminar shear strength of FRPs. Shen et al. [85] incorporated graphene oxide (GO) nanoparticles in GF/EP composites and obtained a 32.7% increase in the ILSS at room temperature and 32.1% in the ILSS at a cryogenic temperature (77 K). Qin et al. [86] coated carbon fibers with GNPs at high density. The results showed that the ILSS increased by 19%. Other fillers have also been considered. For instance, Haro et al. [87] incorporated micro- and nano-powders of alumina, gamma alumina, silicon carbide, colloidal silica, and potato flour into Arall FML (Kevlar-epoxy/aluminum fiber metal laminate), all at around 44 wt % concentration. The ballistic impact results showed that the aluminum powder led to the most optimal impact energy absorption capacity. In addition, the authors highlighted the improvement in fiber/metal bond attained by incorporation of the nanoparticles.

### 2.2. Enhancement of Interlaminar Fracture Strength and Toughness

Delaminations in FRPs or FMLs originate mainly from loads causing mode I or mode II or mixed-mode fracture. It is therefore of paramount importance to report the enhancements that could be gained in the mechanical properties by various means, in order to mitigate delamination of FRPs and FMLs. Various authors have demonstrated that the addition of nanomaterials leads to improved interlaminar fracture toughness (ILFT) and strength (ILFS) [88,89,90,91,92]. For instance, Eskizeybek et al. [54] succeeded in obtaining an impressive 100% improvement in mode I ILFT by spray-coating CNTs onto carbon fibers that were used to construct a laminate, prior to the impregnation of the fibers with an epoxy resin. The superior surface adhesion obtained via spray coating was shown to be the cause of the resulting high ILFT. In fact, no interfacial failure was observed between the fibers and matrix, and only cohesive failure of the matrix itself was reported, which is, as mentioned previously, the desired type of failure. Siegfried et al. [93] showed that CF/EP composite specimens with addition of CNTs performed better under impact due to their improved ILFS compared to the unmodified specimens. However, higher delamination density was observed, which was attributed to the fact that the CNTs made the resin more prone to cracking; in other words, the nanoparticles acted as a stress concentrator under compression loading.

It should be noted that the effectiveness of inclusion of CNTs has not always been so positive. Wichmann et al. [84] observed a 16% increase in the ILSS of their GF/EP with the addition of 0.3 wt % of transversally oriented CNTs; however, the fracture toughness values (G_Ic_ and G_IIc_) were not enhanced. Directly related to mitigation of delamination propagation, Bortz et al. [94] showed that the addition of GNPs led to an increase of 28–63% in the stress intensity factor K_Ic_, a parameter that controls crack propagation.

Concerning fracture mitigation, GNP nanoparticles have proven to be more effective than CNTs. For instance, Rafiee et al. [95] observed a 53% increase in mode I ILFT and a 126% increase in the mode I ILFS of epoxy nanocomposites, using as little as 0.1 wt % of GNPs, compared to 20% and 66%, respectively, when MWCNTs were included. In another study, Rafiee et al. [96] also showed the superiority of GNP as an effective enhancer of mode I fracture toughness compared to nanoclay. A similar trend was observed by Chandrasekaran et al. [97], who demonstrated that inclusion of thermally reduced graphene oxide (TRGO) led to better performances in comparison to GNPs. The higher improvement was attributed to the presence of functionalities for the TRGO particles that proved to have enhanced matrix/particles bonding interaction. Another toughening process was also demonstrated by the authors, which entailed separation of the graphitic layers of the particles. The graphitic layers are held together by a van der Walls force, which is very weak compared to the other intermolecular interaction forces. Thus, some energy is dispersed in the separation process instead of matrix cracking or particle/matrix debonding.

**Figure 4:**
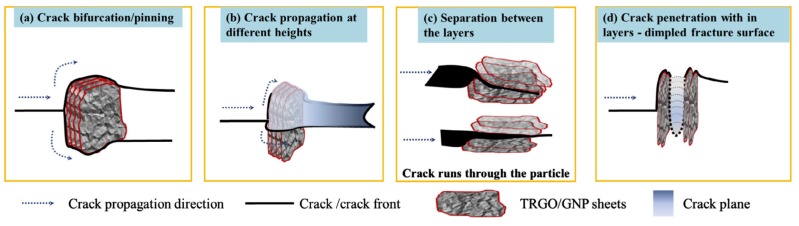
Schematic on the interaction of crack front with GNP/TRGO particles [97] (Reprinted from Compos. Sci. Technol., 97, Chandrasekaran, S.; Sato, N.; Tölle, F.; Mülhaupt, R.; Fiedler, B.; Schulte, K. Fracture toughness and failure mechanism of graphene based epoxy composites, 90–99, (2014), with permission from Elsevier).

Figure 4 shows the different cracking mechanisms, including the separation of the graphitic layers. Ahmadi et al. [98,99] also highlighted the benefits of functionalization of GNP particles on the fracture toughness enhancement for all modes I, II, and III of GF/EP laminates. The authors studied various functionalization agents: NH_2_, graphene oxide, and glycydyloxypropyl-trimethoxysilane (G-Si), and demonstrated that NH_2_ and G-Si yielded the best results, with significant improvement of all three modes of fracture toughness values.

Enhancement gained in fracture toughness by inclusion of NPs is not limited to polymers. Walker et al. [100] introduced 1.5 vol % GNP particles in bulk silicon nitride ceramics, which is even more brittle than thermoset polymers, and obtained 235% improvement in the fracture toughness of the ceramic. Crack deviation was shown to be the mechanism accommodating the observed dramatic increase. 

In addition to the type of NPs, the uniform dispersion of NPs has been shown to significantly affect the expected enhancement in the properties of the mix. Tang et al. [101] demonstrated that the same amount of TRGO nanoparticles (0.2 wt % in their case) could become twice more effective when homogeneously dispersed in the matrix. In the poorly dispersed case, an improvement of 24% in K_Ic_ value was reported, while the improvement was of 52% for the case when particles were uniformly dispersed. To achieve such a high degree of dispersion, the authors combined sonication and a planetary ball mill.

From a microscopic point of view, Mahmood et al. [102] studied the interfacial shear strength between epoxy resin and graphene oxide (GO) coated glass fiber. Their results showed that the coating improved fiber/matrix interface strength by 200%. It is believed that this approach could also improve the metal/FRP interface strength of FMLs.

The effect of other types of nanoparticles on fracture toughness and strength has also been investigated. Zeng et al. [103] examined the mode I ILFT of CF/EP laminates reinforced by soft rubber NPs and rigid silica-NPs (8 wt % and 12 wt %, respectively). They considered the effect of the individual type of NPs, as well as their mixed status. Rubber NPs proved to be 2.5 times more effective in improving the ILFT than the silica NPs. Moreover, by combining both NP types, the margin of improvement fell in between the margins obtained by using the individual NP types. Backed by scanning electron microscopy (SEM) analysis of the delaminated surfaces, the authors attributed the improvement of interlaminar toughness to the cavitation of nano-rubber particles, void growth, and debonding of nano-silica from the epoxy matrix. However, they mentioned that the hybrid effect of nanoparticles needed further investigation. Hsieh et al. [104] also mixed rubber and silica nanoparticles, and observed that the inclusion of rubber NPs led to higher fracture toughness than could be attained by silica NPs. Other similar studies regarding nano-silica particles can be found in references [105,106,107,108,109,110,111,112,113].

Alumina nanoparticles have also been used as effective reinforcement. Zunjarrao et al. [114] and Singh et al. [115] observed an increase in mode I fracture toughness of epoxy and polyester resins with the addition of alumina NPs to the resins; they also investigated the effect of particle size. While the first group of authors reported a greater crack initiation toughness when larger (i.e., micro-size) particles were used, the second group reported an opposite trend, with the smaller particles yielding higher toughness values. As for the carbon-based nanoparticles, the importance of functionalization for a stronger bond between particles and matrix was demonstrated by several researchers (e.g., [116,117]). Finally, the improvements in fracture toughness gained by the use of other NP types in various matrices have also been reported in the literature. The use of nanoclay [118,119], halloysite [120], or the less commonly used vanadium molybdenum [121] are some of the examples.

As mentioned in a previous section, another promising approach that has yielded substantial improvement in both mode I and mode II ILFT and ILFS is the use of the interleaving approach. With this approach, polymer NPs that can eventually be mixed with other components are placed in between composite plies. The incorporation of nanoparticles in the fabrication of these interleaving layers has been shown to enhance their properties, yielding improved ILFT and ILFS. Note that most of the reported research concerning this specific topic is, however, quite recent (started a few years ago).

Ning et al. [122,123] interleaved a carbon black/epoxy layer (with a particle content of 15 g/m^2^) between CF/EP plies, and obtained an improvement of 50.3% in mode I fracture toughness and around 140% improvement in mode II toughness. Zhou et al. [124] synthetized short carbon fibers with CNTs for interleaving CF/EP laminate plies, and reported a 125% increase in mode I delamination fracture energy in comparison to their non-interleaved FRP. Zheng et al. [125] reinforced a less commonly used polymer, polysulfone, with CNTs and used the mixture for interleaving CFRP laminates. They reported an increase in values of both mode I and mode II fracture toughnesses, with the optimum CNT concentration of 10 wt %. The incorporation of GNPs has also been shown to be very effective for interleaving. For instance, Du et al. [126] used functionalized graphene sheets as interleaving substrate in both GF/EP and CF/EP laminates, and obtained up to 140% increase in mode I fracture toughness. As well, the use of GNPs has been shown to have enhanced the flexural modulus and strength [125] of FRPs, and their potentials in facilitating delamination detection and monitoring have also been demonstrated [126]. Kelkar et al. [117] used functionalized and un-functionalized alumina nanoparticles as interleaf materials, and reported, respectively, 51% and 74% increase in the mode I fracture toughness compared to baseline specimens.

Interleaving layers can be manufactured in ready-to-use sheets as well, thus would be compatible with the hand layup manufacturing technique. They can be effectively used along with prepregs to form FRPs and FMLs in an efficient manner, thus, rendering this technique to be an effective and viable solution for enhancing the interlaminar strength of FRPs. This manufacturing approach eliminates the need for mixing nanoparticles in a resin, and then applying the mixture to a fabric.

### 2.3. Enhancement of Fatigue Resistance

As stated, one of the positive attributes of FRPs is their excellent fatigue resistance compared to metals. However, fatigue resistance of FRPs becomes disadvantaged by the onset of a delamination, which often originates in the matrix or at the fiber/matrix interface. To overcome this issue, various researchers have studied the effect of incorporation of nanoparticles in resins for improving fatigue resistance of FRPs and mitigation of the crack growth rate [94,127,128]. For instance, Zhang et al. [129] obtained a 20-fold increase in the fatigue resistance of epoxy resins by inclusion of as little as 0.25 wt % of CNTs to the resin. They also highlighted the fact that the NPs with a higher length to diameter ratio performed better. Rafiee et al. [95] compared the performances of CNTs and GNPs for mitigating crack propagation under fatigue loading. They showed that inclusion of 0.1 wt % GNPs in an epoxy resin led to a reduction of crack growth rate by up to two orders of magnitude, when tested at a high stress intensity factor (0.6 MPa·m^1/2^), compared to the pristine epoxy. In contrast, their CNT-reinforced epoxy specimens, tested at the same stress intensity factor, did not show any evidence of performance improvements. However, this difference in behavior between GNP- and CNT-reinforced matrix was not as noticeable when the specimens were tested at lower stress intensity factors.

The improvements gained by inclusion of NPs in thermoset polymers have been shown to also hold for reinforcing natural rubber. Yan et al. [55] incorporated graphene into natural rubber and observed that at low fatigue strains, the crack growth was accelerated with the addition of NPs, while the opposite effect was reported for higher strains. The authors suggested this behavior to be linked to the crystallization process. At higher strain rates, crystallization occurs, causing a deviation of the crack path and leading to higher energy dissipation, therefore reducing crack growth. The inclusion of graphene seemed to exaggerate this phenomenon. However, at lower strain rates, the crack propagation mechanism is governed by the coalescence of micro-voids close to the crack tip, whose number is increased by the incorporation of graphene. A more significant increase in the fatigue resistance of a GF/EP composite was obtained by Yavari et al. [130], by directly spray-coating the fiberglass fabric with only 0.2 wt % of GNPs. They observed up to 1200-fold increase in the fatigue life of the composite under flexural bending mode. The authors attributed this elevated improvement to the suppression of the interlaminar crack propagation and of the delamination/buckling of the fiber/matrix interface.

Non-carbon-based NPs have also been shown to be effective in improving the fatigue resistance of composites. Manjunatha et al. [131] incorporated 10 wt % of silica nanoparticles into an epoxy resin and observed an improvement in the tensile fatigue resistance of the resin. They also observed significant improvement of 300–400% in the fatigue life of the GF/EP laminate composite made by the reinforced resin compared against the performance of their baseline laminate. The authors attributed the improvement to two energy dissipating mechanisms facilitated by (i) particle debonding and (ii) the subsequent plastic void growth in the matrix. Improvement in the fatigue life of FRPs with the use of silica NPs was also reported by Blackman et al. [132]. In contrast, when Akinyede et al. [133] incorporated alumina NPs into a matrix, no noticeable improvement in the fatigue life was observed, although, as described earlier, improvement in fracture toughness of resins was attained by inclusion of alumina NPs.

### 2.4. Effects on Delamination

As mentioned in the introduction, several articles have explicitly reported the positive effects of inclusion of NPs in composites to specifically mitigate delamination growth under various loading conditions. Notwithstanding, the opposite effects have also been observed and reported by several researchers. For instance, as mentioned in a previous section, Siegfried et al. [93] incorporated CNTs into the matrix of CFRP plates, and reported improvements in the ILSS and mode II interlaminar strength values, as well as an overall increase in the total impact energy of the specimens. However, the delamination areas after impact of the plates were larger than the plates formed with the neat matrix. The authors attributed the phenomenon to a higher matrix crack density caused by inclusion of CNTs. Avila et al. [56] reinforced glass-epoxy FRP with nanoclay and nanographite particles. Specimens reinforced with nanoclay, subjected to ballistic impact, showed an average of 68% increase in delamination growth on the impacted surface compared to non-NP reinforced glass epoxy specimens. Those reinforced with nanographite particles showed no change, either positive or negative. However, the delamination extent on the non-impacted surface was observed to have increased by 2930% and 557% in specimens hosting nanoclay and graphite NPs, respectively. Slightly better results were reported by Kamar et al. [134], who incorporated GNPs in a GF/EP laminate. They observed a lesser area of delamination on the impacted surfaces of their NP-reinforced specimens compared to non-NP-reinforced specimens, while a larger delaminated area was observed on the non-impacted side. However, improvements were also reported. Yokozeki et al. [135] observed decreased levels of 3% and 1.5% in the delamination areas when 5 wt % and 10 wt % cup-stacked CNT were mixed in the epoxy matrix, respectively, forming their CFRP. Daelemans et al. [136] reported up to 50% reduction in the delaminated area after out of plane impact, compared to neat specimens, for (0/90)_2s_ GF/EP composites interleaved with poly(ε-caprolaptone) electrospun nanofibrous veils.

It should be noted that a clear majority of the experiments that examined the influence of NPs on delamination length were carried out under impact loading, while when characterizing the other properties, the experiments were conducted under a quasi-static rate. Moghim and Zebarjad [137] performed low-strain rate tests and reported an increase in the brittleness of CNT-reinforced matrix with an increased strain-rate. Similar results were obtained by Shadlou et al. [138] using GNP nanoparticles in an epoxy resin. They performed tensile tests with strain rates up to 10 s^−1^, and reported a transition from ductile to brittle behavior in the case of GNP-reinforced resin. Moreover, the stress softening behavior after the yielding point, observed in the case of their neat epoxy, vanished in specimens that included GNPs. However, in both the former cited works, increases in the Young’s modulus and strength of the GNP-reinforced epoxy resins were reported. An increase in the extent of delamination was also reported in the specimens that were tested under higher strain-rates.

### 2.5. Enhancement of Adhesively Bonded Joints

As mentioned previously, enhanced polymer properties could also be beneficial to the performance of ABJs. However, even though the previously cited works can be used to show the potential of nanoparticles in ABJs, it is worth mentioning some of the works that have specifically considered the enhancement of ABJs with incorporation of NPs. An extensive volume of research has been carried out on this topic, with the vast majority of it dealing with carbon-based nanoparticles, but other particles have also been used (e.g., nano-silica [139] or titanium oxide [140]). Note that most of the literature on this topic is fairly recent (from 2014 onward). For a comprehensive review of this subject, the reader is directed to the review paper presented by Shadlou et al. [141]. Here we mention some of the noteworthy works that have been added to the literature subsequent to reference [141].

Sydlik et al. [57] showed that the addition of 1 wt % of functionalized MWCNTs into epoxy adhesive led to an increase of 36% in the lap shear strength, compared to that of the neat adhesive. Wernik and Meguid [142] carried out a comprehensive set of tests to analyse the effect of the incorporation of CNTs into epoxy adhesive. Dog-bone tensile, single- and double-lap shear, and double cantilever beam tests were carried out. The results revealed a 90% increase in the tensile bond strength, 54% in the bond shear strength, and 36% improvement in the energy release rate when the nanoparticles were treated with polyvinylpyrrolidone, a solvent used to improve the dispersion of the particles. Similar results were obtained by Gude et al. [143], who also highlighted that an increase of the extent of delamination resulted from the addition of NPs.

Graphene NPs have also been used as nano-fillers in adhesives. For instance, Gültekin et al. [144] added 1 wt % of graphene to reinforce an epoxy adhesive, which was incorporated to construct double-lap shear specimens with aluminum adherends. Results showed an increase in the failure capacity and ductility of the ABJs. However, greater filler contents led to degradation of the bond capacity, even resulting in a lower capacity than that offered by the neat epoxy. Guadagno et al. [145] also showed that the inclusion of graphene nanoparticles improved the interface strength between the adhesive and epoxy adherends. Mohamed and Taheri [146] studied the effect of GNPs on mode I fracture toughness of GF/EP subjected to thermal fatigue. Their ABJs were subjected to incremental thermal cycles of −35 °C to +45 °C, up to 1000 cycles. They showed the inclusion of GNPs in the adhesive reduced the effect of thermal fatigue (i.e., degradation of the mechanical properties of the resin was reduced). Kubit et al. [147] and Zielecki et al. [148] carried out fatigue peel tests using aluminum and steel adherends, respectively, bonded with MWCNT reinforced epoxy. They observed an increase of both fatigue strength and fatigue life, though more significant improvement was observed in the fatigue life in comparison to the improvement in the strength.

A comparison between the effectiveness of different nanoparticle types in enhancing the load-bearing capacity of ABJs was also carried out by a few investigators. For instance, Soltannia and Taheri [149] compared the level of enhancement gained by inclusion of CNT, CF, and GNP nanoparticles in a widely used inexpensive epoxy resin. The neat and reinforced resins were used to fabricate single-lap ABJs with unidirectional CF/EP and GF/EP adherends. The ABJs were subjected to tensile loadings applied at various loading rates (from 1.5 mm/min to 2.04 × 105 mm/min). Their results indicated that the resin reinforced with 1 wt % of GNP yielded the greatest improvements. Jojibabu et al. [150] also obtained greater strengthening of the efficiency in their ABJs when GNPs were included in an epoxy adhesive, compared to inclusion of SWCNTs and carbon nanohorns in the same epoxy. Ayatollahi et al. [151] incorporated carbon- and mineral-based nanoparticles (i.e., MWCNTs and nanosilicas, respectively), in an epoxy adhesive. Addition of silica resulted in the highest shear strength and elongation values in their ABJs. Other works concerning silica nanoparticles can be found in [139,152].

The effect of highly conductive nanoparticles (mainly carbon-based) on the electrical conductivity was also subject of several studies. The main goal of such studies has been to develop an effective and reliable method for diagnosing and monitoring damage within adhesives used to form ABJs. Mactabi et al. [153] demonstrated the possibility of monitoring the integrity of ABJs under fatigue loading by inclusion of CNTs within adhesives. They showed that a change of 10% in electrical conductivity indicated that 60–90% of the fatigue life had been reached. Kim and Choi [154] analyzed the effect of five different techniques for dispersing CNT NPs within an adhesive. The goal was to evaluate the degree of conductivity of the bond region produced by the dispersion techniques, as well as the overall efficiency of the techniques by evaluating the strength of the resulting ABJs. The authors reported that the only technique that led to enhancement of joint strength was the sonication technique; however, the resulting adhesive produced the lowest level of defect detection ability. All the other techniques resulted in joint capacities lower than that exhibited by the neat adhesive. Jakubinek et al. [155] used SWCNTs to reinforce an epoxy adhesive. While the ABJs formed with the adhesive hosting 1 wt % SWCNTs exhibited a 30% increase in the peel strength compared with the capacity of joints made with a neat epoxy, the lap-shear strength of the joint was degraded by 10–15%, and the improvement of electrical conductivity was lower than that obtained from the theoretical analysis. However, they managed to attain conductivities as high as 10^−1^ S/m after applying post-manufacturing electrical treatment. Kang et al. [156] also reported a decrease in the static lap-shear strength of their ABJs when CNT NPs were used to reinforce their adhesive. However, they observed an increase in their bonded joints’ fatigue strength. Moreover, the inclusion of the nanoparticles allowed more effective monitoring of crack initiation and propagation.

The mechanisms that are responsible for enhancing the mechanical properties of ABJs have been identified to be similar to those presented earlier in this paper, when delamination mitigation of FRPs was discussed (i.e., crack bridging and crack deflecting; see [57,145,151,157]). As also discussed earlier, NPs contents above a certain wt % have also been found to cause degradation of the adhesives’ mechanical properties, mainly due to agglomeration of the particles. Moreover, some authors [151,157] reported a switch from interfacial to cohesive failure mode of the joint, which is, as mentioned earlier, the most suitable type of failure, because the mode is more energy demanding. Unfortunately, however, a certain level of variability in the reported results has been noted. A big factor responsible for the reported results inconsistency is believed to be due to the techniques used in dispersing NPs within adhesives. Indeed, adhesive type and NPs’ type and content (wt %) also contributed to the results’ variability. It is strongly believed that the use of the most effective dispersion technique would result in more reliable ABJs, whose response could be reliably predicted by appropriate analytical methods, thereby broadening the use of this effective joining technique.

### 2.6. Other Notable Effects

In this section, some of the other notable effects that have been observed to result from the incorporation of NPs in FRPs and ABJs will be discussed. Firstly, gains in stiffness and strength have been reported by several authors [95,125,135,137,158]. Secondly, augmentation of the electrical conductivity, experienced mainly with carbon-based nanoparticles, has also been reported by some authors [84,101]. These enhancements could be quite beneficial in applications where a certain level of conductivity is required (e.g., in some specific aerospace components used to protect the aircraft from a lightning strike), or for strain-stress, damage and crack evolution monitoring [159].

In addition to the above, the augmentation of the glass transition temperature of polymer matrices has also been reported [101], which is beneficial for enabling the use of FRP at higher temperatures. Moreover, the enhancement in resistance to combustion by inclusion of nanoclay and GNP NPs in FRPs has been highlighted by references [58,160,161,162,163,164,165].

## 3. Numerical Modelling of Delamination in FRPs and ABJs

As the complexity of materials and systems increases, both research and industry sectors rely increasingly on numerical simulations to understand the behaviour of materials and structural systems created by them. Numerical approaches allow one to investigate the influence of various parameters on the response of structural systems much more efficiently compared to conventional experimental investigations. As such, one can conduct parametric studies, by which the influences of a large number of parameters could be investigated in an effective and efficient manner. One could even obtain certifications for certain structural systems, without resorting to expensive experiments, thus saving time and money (many of the aerospace and marine systems fit into this category).

The most extensively used numerical approach is the finite element method, which will be the main focus of the literature review outlined in this part of the manuscript. It should be noted that the numerical approaches used for studying delamination in FRPs would also be applicable for investigating the performance of ABJs. In addition to the works cited throughout this section, the reader is urged to refer to a recent thorough review conducted by Bernardo et al. [166], which specifically considers modeling and simulation techniques used in characterizing nanoparticle reinforced polymers.

### 3.1. Cohesive and Extended Finite Elements (XFEM) Modeling Techniques

When the failure path is known and limited to taking place within the adhesive or resin layers, then one of the most effective means to model the system would be by incorporating the cohesive elements in conjunction with an appropriate cohesive law. This combination takes into account the progressive separation of the bonded layers, and can mimic the damage occurring with the cohesive layer, which could eventually lead to the complete decohesion of the layers. With this approach, the interface between any two components (which could be plies in a laminated composite, or metallic and FRP plies in a FML, or simply two adhesively bonded adherends), could be simulated with these special elements [167,168,169,170].

Yelve and Khan [171] used cohesive zone modeling (CZM) for simulating the separation of two bonded aluminum adherends in the form of a double-cantilever beam, and stated that this simulation approach could also be used to simulate the response of interfaces in an FRP system. May et al. [172] used CZM for the simulation of debonding in a T-joint. This approach has been used to model the effect of fatigue [173,174,175,176], moisture [177], and thermal effects [178] on various interfaces with success.

In cases where a crack’s initial location and/or its propagation path are unknown, another more recently developed simulation technique, namely the extended finite elements (XFEM), could be used to conduct the analysis. XFEM is essentially a general term used to refer to any method that consists in enriching the finite element formulation in order to allow for discontinuities to develop within a series of elements, thus enabling modeling of crack initiation and propagation [179,180,181,182]. This method has been successfully applied for modeling of crack initiation and propagation in composite materials [183]. Another example is the work of Motamedi and Mohammadi [184], who enriched previously available XFEM formulations to allow for a more accurate solution around the tip of a propagating crack in an orthotropic medium. More specific to modeling of delamination, one can cite the work of Wang and Waisman [185], who used a discrete damage zone within the extended finite element method to simulate delamination of laminated composites, that allowed the representation of both interfacial debonding and bulk cracking, all in a mesh-independent fashion.

### 3.2. Various Scale Modeling Techniques

In addition to the above-mentioned modeling techniques, the distinction between the different modeling scales that have been introduced in the literature could also be discussed. In some cases, the computational power available for carrying out simulations in very fine scale could be limited. Therefore, depending on a given component’s size and the mechanisms that one would want to study, different modeling scales could be considered. The modelling scales are categorized under macroscopic, microscopic, and molecular, or a combination of the three, commonly referred to as multi-scale modeling. Note that these modelling techniques are discussed in reference to nanoparticle-reinforced media.

#### 3.2.1. Macroscopic Scale Modelling

In the macroscopic scale, a component’s size is adequately large, so that microstructure consideration would not be necessary. In such a case, only the homogenized properties are used, and the sought output would be that describing the global response of the system in terms of stress, displacements, energies, etc. For example, when modeling the response of an NP-composite system, the nanoparticles would be integrated into one or more constituents; in other words, the nano-particles themselves are not explicitly modeled. Instead, their effect is taken into account by varying the material properties of the material hosting them. This approach is suitable for modelling the response of real size components and structures, ranging from a few millimeters long coupons to full-scale components.

For example, Jiang [186] studied the decohesion mechanism and properties of CNTs reinforced dry adhesive, using cohesive elements to simulate the interface of the constituents. The material properties were extracted from micromechanical studies (this will be discussed in the following paragraph). Grail et al. [187] used CZM to model the effect of interleaving in CRFPs, with a focus on the optimization of the interleaf layers to increase strength under uniaxial tensile loading. The authors showed that by carefully placing artificial delaminations in the specimen, the stress concentration could be reduced, leading to an increase in the overall strength. The effect of the incorporation of nanoparticles in the interleaf layers could easily be taken into account using this approach, hence further optimizing the interlaminar decohesion strength. Asaee et al. [188,189] used CZM to model the effect of NH_2_ functionalized GNP particles on the impact resistance and delamination-buckling behavior under static loading of a newly developed 3D fiber-metal laminate, demonstrating that good agreement with experimental results could be obtained by appropriately calibrating the material parameters, thus accounting for the presence and influence of the nanoparticles.

#### 3.2.2. Microscopic Modeling Scale

**Figure 5:**
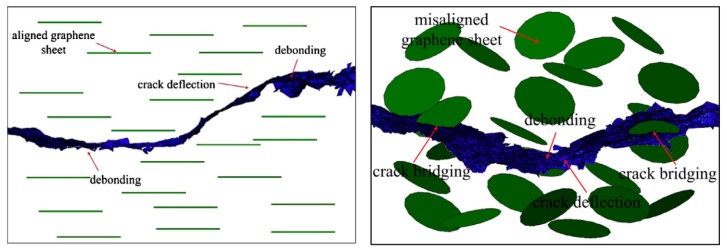
Crack morphology in an aligned and random model [192] (Reprinted from Comput. Mater. Sci., 95, Dai, G.; Mishnaevsky, L.J., Graphene reinforced nanocomposites: 3D simulation of damage and fracture, 684–692, (2014), with permission from Elsevier).

On the other side of the analysis spectrum is the microscopic scale modeling scheme, by which the actual microstructure of the component is included in the model. For instance, in the case of modelling the response of NPs included in a resin, the NPs would be explicitly modeled. As can be expected, due to the relatively extremely small size ratio of NPs and their surrounding matrix, and computation limitations, such models would be usually of only a few microns in size. This approach can be used to obtain the homogenized properties of the mix, better understanding of the microstructure of the constituents (e.g., the crack propagation mechanism in the media), study of the bonding interaction between the particles and the matrix, and conducting parametric studies on the shape, aspect ratio, and special distribution of NPs and void effect. Note that due to the microscale nature of the analysis, the delamination between the constituents of the system cannot be modeled, but understanding of the microscopic mechanisms helps the analyst to gain a better perspective of the parameters that critically influence the crack propagation, ILSS and fracture toughness, and energy release rate. Safaei et al. [190] modeled the debonding of GNP flakes from the surrounded matrix. They used the CZM to model the interface between the flakes and the matrix. Similarly, Guo and Zhu [191] studied GNP/matrix interaction by coupling the CZM and shear lag model, with the cohesive elements used to model matrix/flakes interfaces. They successfully modeled three possible status of the system: (i) the flakes remaining bonded to the matrix; (ii) the flakes damaged by the applied load; and (iii) the flakes’ debonding from the matrix under the applied load. Dai and Mishnaevsky [192] simulated the propagation of a crack in a GNP/matrix media, using XFEM. The results, illustrated in Figure 5, show the different mechanisms that governed the crack propagation, for both oriented and non-oriented particles. Crack bridging, debonding, and crack deflection mechanisms were captured. The simulated results showed good agreement with their experimental results. The authors highlighted the difference in crack propagation between oriented and non-oriented graphene sheets, demonstrating the high influence of the particles’ orientation on the mechanical properties and crack propagation. It was observed that the aligned GNPs yielded greater mechanical strength and stiffness compared to those produced by misaligned GNPs. Also, clustering of the sheets led to degradation of the properties.

Moreover, many researchers have also investigated the shear debonding of NPs. A few studies, however, have considered the debonding of graphene under the peeling mechanism. One of the notable studies is that by Jia and Yan [193], who modeled the mechanism with the use of cohesive elements modelling the particle/matrix interface.

From the modeling perspective, as briefly stated earlier, the very small size of the nanoparticles makes it tedious to manually represent their random features, such as their orientation, agglomeration, aspect ratio, and average content, as well as limiting the modeling to very small volumes. Automatization of the required model generation would be a viable partial solution to this problem, and it can be achieved with two different approaches. One such approach, as tried by Sheidaei [194], entails using 3D SEM micro-scanning of the NP-reinforced medium, followed by the automatic generation of an FE mesh by mapping the image. In this approach, a statistical analysis of the dispersion of the particles is done first from the image, and then, the data is used to generate the corresponding finite element model. With this technique, the authors could successfully model Halloysite clay nanotube and exfoliated GNP enhanced polypropylene composites. The results reported by the authors show the potential of the noted approach, despite their statement that appropriate modifications of the method would be necessary to obtain fully reliable results. Wang et al. [195] used a different approach; their approach involved the development of an algorithm that could automatically generate the dispersion map of nanoparticles in a medium by specifying certain parameters, such as shape, aspect ratio, dispersion content, and orientation. This approach was proven to be very efficient for carrying out parametric and optimization studies. The micromechanical approach has a great potential for studying delamination mechanisms, since a thorough understanding on the crack propagation will enable efficient optimization procedures.

#### 3.2.3. Molecular Modeling Scale

An even smaller-scale modeling approach than micro-modelling has also been tried, which is referred to as molecular scale modelling. In this scale of modelling, the different components are not treated as material continuums, but as the name indicates, their molecular structures are modeled. It should be noted that the inter-molecular interaction forces that govern the overall behavior of such minutely-scaled models are different from those considered at larger scales. Therefore, in addition to FE modeling, the molecular dynamics (MD) approach, which is based on the concept of continuum media, is also used. The former approach entails modelling of each atom as a distinct particle, as well as modelling the interaction between the atoms. Nevertheless, this modeling approach could facilitate a thorough understanding of the mechanisms that govern the behavior of the system at the macro-scale; however, in practice, it could only handle a very small representative volume of a given composite.

A good representative example can be seen in that of Awasthi et al. [196]. In this work, the authors studied the opening and sliding mode behavior of CNT/polymer interaction by modeling the system at a molecular level, under different boundary conditions. To simplify the study, a portion of the surface of the CNTs was modeled as a graphene sheet, interacting with polymer molecules. Results obtained by their model revealed that the interaction between the graphene and polymer was stronger than the polymer’s chains, since breakage of the polymer chains was observed to be the most distinct failure type, while debonding of the polymer from the graphene NPs was not observed. On the same principle, Wernik and Meguid [197] used an atomistic-based continuum approach to model the interaction between adhesive and CNTs particles in an ABJ, taking into account the non-linear behavior of the medium. The authors used the nodes of the FE model to represent the atoms, and used truss elements to construct the CNT structure, and solid elements for modelling the matrix structure. Moreover, truss elements were used for representing the interaction mechanism between the atoms of the CNT and the matrix. Simulations showed the improvement in stiffness and strength that could be attained by inclusion of the NPs.

Odegard et al. [198] simulated the behaviour of silica nanoparticle/polyamide composites formed with various NP/matrix interface treatments, using both MD and FE modeling techniques. MD was used to extract the equivalent mechanical properties of the particles and matrix (i.e., by using the molecular weight of the chains, the mechanical properties consistent with experimental results could be obtained), as well as the interaction strength. These values were then used in an FE model consisting of a representative volume, which included a few particles, matrix, and their interface zone. The size of the nanoparticles was progressively increased in order to identify the viable size at which the Mori-Tanaka homogenization model could be considered admissible (note the model does not take into account the interface properties). It was found that the Mori-Tanaka model would be admissible when the particles had a minimum radius of 0.1 μm. Modeling at this scale would be particularly useful when one wants to study the interface interaction between different materials, leading to great improvements in their overall strength and delamination resistance. For example, it can be applied to investigate the bonding mechanism between various substrates with an adhesive.

#### 3.2.4. Multiscale Modeling Approach

In addition to the approaches briefly discussed above, there have been some studies that have used the multiscale modelling approach, which should be mentioned here, even though they did not involve the study of the influence of nanoparticles. The high potential in attaining improved delamination mitigation and bonding strength with the use of such an approach merits its brief review.

**Figure 6:**
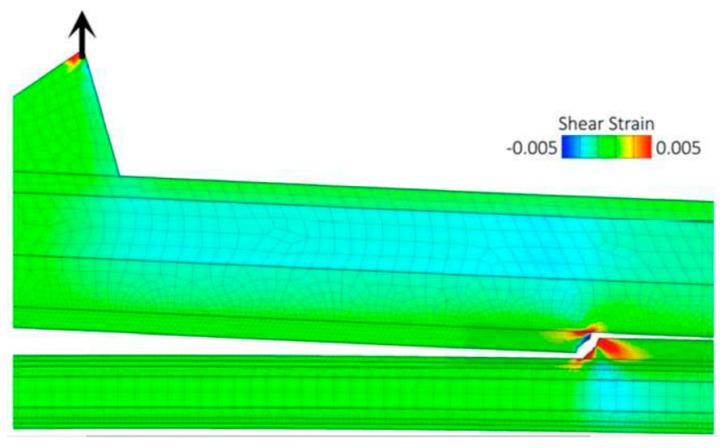
The central region of the specimen where delamination moves from plies interface into a ply [199] (This figure has been reprinted by permission from the Proceedings of the American Society for Composites: Thirty-first Technical Conference, 2016. Lancaster, PA: DEStech Publications, Inc.).

**Figure 7:**
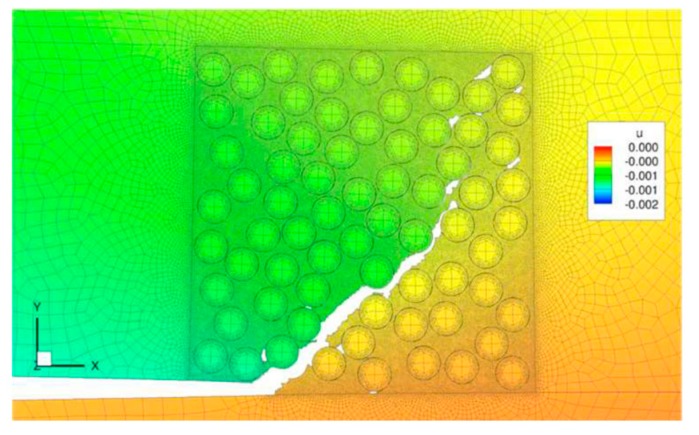
Deformed image of the damage evolution simulated by the micro-scale model. The elements in which the cohesive zone damage variable exceeded 0.975 have been removed (displacements are in μm) [199] (This figure has been reprinted by permission from the Proceedings of the American Society for Composites: Thirty-first Technical Conference, 2016. Lancaster, PA: DEStech Publications, Inc.).

Mollenhauer et al. [199] simulated the delamination of CF/EP by coupling micro- and macro-scale approaches, as well as cohesive and XFEM. The combination of cohesive elements and XFEM allowed simulation of the transition in the failure mechanism, from delamination of the plies to failure running within the plies (cf. Figure 6). In this work, microscale modeling was used at the point of bifurcation to reproduce the crack propagation between the fibers with high fidelity (Figure 7).

With reference to the figure, the delamination initiates between the 0° plies (situated below the visible crack) and 90° plies (located above the crack). The crack then deviates from its original path, propagating into the 90° plies. At this stage, the crack changes its course again, once it encounters the other 0° plies, where it continues and causes the final delamination failure of the composite. As can be seen, the system represented in Figure 7 is the microscale representation of a portion of the model shown in Figure 6, where the crack deviation is visible. An extension of this strategy can examine the influence of inclusion of nanoparticles on crack propagation. Following the same principle, one can, for instance, assume a nanoparticle is occupying the zone highlighted in red in the figure, and apply the properties of an NP to that portion of the model, thereby examining the effect of nanoparticles on the crack deviation process.

Along the same lines, Hadden et al. [200] coupled molecular and micro-scale approaches to model behaviour CFRP composite reinforced with GNPs. The effects of particle directions, volume fraction, and dispersion were studied. As was shown by the experimental observations presented in the previous sections, the authors could demonstrate that the particles did not significantly influence the in-plane properties of the composite, but the through-thickness properties were improved. The molecular-scale analysis also showed the effect of the number of stacked graphene sheets: a higher number leads to a stiffer behavior, under both tensile and shear loading states.

## 4. Summary and Conclusions

Fiber-reinforced polymers have been shown to have excellent mechanical properties. However, depending on the polymer type used in their formation, they exhibit some limitations. In general, between the two widely used polymers in structural applications, thermoset resins are relatively stiffer and stronger, and are more cost-effective than their thermoplastic counterparts. However, thermosets are more brittle than thermoplastics, and therefore, prone to cracking, and if used to form FRPs, the resulting laminates would be more susceptible to delamination. Addition of nanoparticles to both types of polymers has been shown to be a viable and effective method to overcome the aforementioned issues. In particular, NPs have been shown to effectively improve the fracture toughness of thermoset resins, and enhance the out-of-plane properties of the laminate composites, and fiber-metal laminates formed by such reinforced resins. The same approach has been shown to render more superior adhesively bonded joints.

The focus of this review paper has been to highlight the role of NPs in fracture toughness and fatigue endurance of polymers, in turn improving the interlaminar shear strength (ILSS) of the laminate composites and adhesively bonded joints created by such reinforced resins. As seen, a significant amount of works has been conducted on the noted topics, leading to a large number of publications. The conclusion of these works can be summed up in stating that the inclusion of a small amount of appropriate nanoparticles to a polymer matrix can lead to significant improvements in the mechanical properties of the hosting matrix, so long as the NPs have been dispersed uniformly within the matrix. A few works have also reported the detrimental influence of this strategy, by observing degradation in delamination resistance of FRPs, especially under impact loading, when the matrix ductility was actually reduced due to NP content above the appropriate threshold limit.

This review article also provided a summary of the various numerical approaches and strategies that have been developed and used by various investigators to simulate the influence of NPs within a matrix. The different scale modeling approaches, which enabled modeling of relatively large to microscopic scale materials were also discussed. The efficiency of conducting a numerical parametric study was also highlighted, which enables one to gain a thorough understanding of the involved complex nonlinear mechanisms.

Nanoparticles type, dispersion, aspect ratio, and size have been shown to affect the behavior of the host matrix. One can conclude from the results published in the literature that amongst all commonly used nanoparticles, the functionalized graphene nano-platelets could optimally improve performance of thermoset resins in the most cost-effective manner. Notwithstanding, the presented review also highlights the variability noted in the reported results. The difficulty in attaining uniform dispersion of nanoparticles and a lack of understanding of some of the micro-mechanisms governing their general behavior, are postulated to be responsible for the noted discrepancies in the published results. Additional works in this area are therefore warranted in order to better understand the effect of nanoparticles on the performance of composites made by them, especially with a focus on their performance under various strain rates. In addition, our survey indicates that there is a clear lack of studies that consider the influence of NP-reinforced resins on aging and moisture absorption.

## Figures and Tables

**Table 1:** Mechanical properties of some commonly used thermoset and thermoplastic resins and their costs [5].

Resins	Density *ρ* (kg/m^2^)	Elastic Modulus *E* (MPa)	Shear Modulus *G* (MPa)	Poisson Ratio *ν*	Tensile Strength *σ_ult_* (MPa)	Elongation *ε* (%)	Coefficient of Thermal Expansion *α* (°C^−1^)	Price 1993 ($/kg)
Epoxy	1200	4500	1600	0.4	130	2 (100 °C)	0.2	6 to 20
						6 (200 °C)		
Phenolic	1300	3000	1100	0.4	70	2.5	0.3	-
Polyester	1200	4000	1400	0.4	80	2.5	0.2	2.4
Polyether-ether-ketone (Peek)	1300	4000	-	-	90	50	0.3	96

**Table 2:** List of treatments for modification of metal surfaces, as reported in [59]. Note the reference numbers correspond to the one in the original document.

Treatments	Nature of Treatments	Reference
Grit blasting	Mechanical	26–29
Chromic-sulphuric acid (CAE)	Acid etching	30,33
Sulfo-ferric acid (P2)	Acid etching	31
Forest Product Laboratory (FPL)	Acid etching	32
Alkaline	Etching	25,30
Chromic acid anodizing (CAA)	DC-anodizing	46
Phosphoric acid anodizing (PAA)	DC-anodizing	23,34,36
Sulphuric acid anodizing (SAA)	DC-anodizing	36,45
Boric-sulphuric acid anodizing (BSAA)	DC-anodizing	24
Phosphoric acid anodizing (AC-SAA)	AC-anodizing	36
Sulphuric acid anodizing	AC-anodizing	36
Silane	Coupling/oxidation	28,47,49–52
Sol-gel	Coupling/oxidation	53–57
Excimer laser texturing	Mechanical	54,58–62
Plasma sprayed coating	Ablation/oxidation	35,63–67,69
Ion beam enhancement deposition (IBED)	Ablation/oxidation	62,68

## Abbreviation and Acronyms

ABJ	adhesively bonded joint
CF	carbon fiber
CF/EP	carbon fiber-epoxy composite laminate
CFRP	carbon fiber reinforced plastic
CNT	carbon nano-tube
CNF	carbon nano-fiber
CSCNT	cup-stacked carbon nano-tube
CZM	cohesive zone model(ing)
FML	fiber-metal laminate
FRP	fiber-reinforced polymer composite
GF/EP	glass fiber-epoxy composite laminate
GNP	graphene nano-platelet
GO	graphene oxide
ILFS	interlaminar fracture strength
ILFT	interlaminar fracture toughness
ILSS	interlaminar shear strength
MD	molecular dynamics
MWCNT	multi-walled carbon nano-tube
NP	nanoparticle
SWCNT	single-walled carbon nano-tube
TRGO	thermally reduced graphene oxide
Note: “s” following above acronyms makes them plural	
